# Mechanistic Investigation of Adociaquinone and Xestoquinone Derivatives in Breast Cancer Cells

**DOI:** 10.3390/md23120464

**Published:** 2025-12-02

**Authors:** Yu-Dong Zhou, Fakhri Mahdi, Nicholas M. Nagle, Mika B. Jekabsons, Dale G. Nagle

**Affiliations:** 1 National Center for Natural Products Research, School of Pharmacy, University of Mississippi, Oxford, MS 38677-1848, USA; 2 Department of Biomolecular Sciences, School of Pharmacy, University of Mississippi, Oxford, MS 38677-1848, USA; 3 School of Engineering, University of Mississippi, Oxford, MS 38677-1848, USA; 4 Department of Biology, College of Liberal Arts, University of Mississippi, Oxford, MS 38677-1848, USA

**Keywords:** adociaquinone, xestoquinone, HIF-1, mechanism of action, respiration, redox cycling, stress response, breast cancer cells

## Abstract

Xestoquinone derivatives isolated from marine sponges exhibit a range of bioactivities, including the inhibition of HIF signaling, mitochondrial function, and tumor cell proliferation. Mechanistic investigation suggested that 14-hydroxymethylxestoquinone (**1**) acts as a protonophore. Although adociaquinones A (**5**) and B (**6**) each stimulated cellular oxygen consumption, neither affected mitochondrial membrane potential. Cell-based respiration studies revealed that adociaquinones restored sodium azide-stalled oxygen consumption and ascorbate enhanced this response, suggesting ascorbate-supported redox cycling as a possible mechanism by which adociaquinones suppress HIF and tumor cell proliferation. These xestoquinone derivatives activated cellular stress response pathways that inhibit protein translation by phosphorylating key regulatory proteins (i.e., eIF2α, eIF4E, and eEF2). Further, thiol-reducing agents NAC and DTT attenuated the monosubstituted xestoquinone derivatives’ efficacy to inhibit HIF signaling, suggesting a potential mechanism of action that involves sulfhydryl modification.

## 1. Introduction

Multicellular organisms have evolved tightly regulated oxygen delivery systems to ensure oxidative phosphorylation-dependent conversion of the energy stored in nutrients into ATP. In the human body, high levels of oxygen (hyperoxia) can cause oxygen toxicity and low oxygen levels (hypoxia) can trigger an energy crisis that culminates in the death of cells with a strong reliance on oxidative phosphorylation [1]. For neoplastic disorders, hypoxia is a signature feature of the tumor microenvironment. The oxygen-regulated transcription factor hypoxia-inducible factor-1 (HIF-1) promotes tumor cell survival and adaptation to hypoxic conditions [2]. Composed of an oxygen-labile α-subunit and a constitutively expressed β-subunit (HIF1β/ARNT), HIF-1 is induced and activated by hypoxic conditions [3]. Hundreds of target genes regulated by HIF-1 participate in nearly all key cellular processes [2]. A large body of evidence from preclinical and clinical studies supports HIF-1 as an important molecular target for drug discovery [2].

During our screening campaign for natural product-derived inhibitors of HIF-1 signaling, seven xestoquinone derivatives were isolated from an extract of the marine sponge *Petrosia alfiani* de Voogd & van Soest (Petrosiidae) and their HIF-1 inhibitory activities were characterized [4]. In a human breast cancer T47D cell-based reporter assay, low micromolar IC_50_ values were observed. Further, adociaquinones A and B exhibited >20-fold selectivity towards the inhibition of HIF-1 activation by chemical hypoxia in comparison to that induced by low oxygen. Chemical hypoxia can be induced by iron-chelating agents (e.g., deferoxamine, 1,10-phenanthroline, etc.) and transition metals (e.g., cobalt chloride), while a range of pathological conditions (e.g., tumor hypoxia, ischemia, etc.) can yield hypoxia. Mechanistic investigation revealed that the HIF-1 inhibitory xestoquinone derivatives increased cellular oxygen consumption. While active compounds such as 14-hydroxymethylxestoquinone function as a protonophore that dissipates mitochondrial inner membrane potential and stimulates respiration, adociaquinones did not affect mitochondrial membrane potential [4]. Previous studies reported enzyme inhibitory activities for adociaquinones A and B [5,6,7]. Building on the hypothesis that adociaquinones exert their biological activities through redox cycling, T47D cell-based oxygen consumption studies were conducted in the presence of the cytochrome c oxidase inhibitor sodium azide. Adociaquinones A and B each initiated sodium azide-stalled oxygen consumption and ascorbate exacerbated this restoration, suggesting that adociaquinones function through redox cycling. Mechanistic investigation revealed that these compounds trigger a cellular stress response that suppresses protein translation by phosphorylating key factors that include the initiation factors eIF2α and eIF4E, and the elongation factor eEF2. These studies found that *N*-acetylcysteine inhibited the cellular stress response induced by the most active 15-hydroxymethylxestoquinone, suggesting the importance of redox homeostasis for the active xestoquinone derivatives to exert their bioactivities.

## 2. Results

### 2.1. Tumor Cell Oxygen Consumption Studies

**Figure 1:**
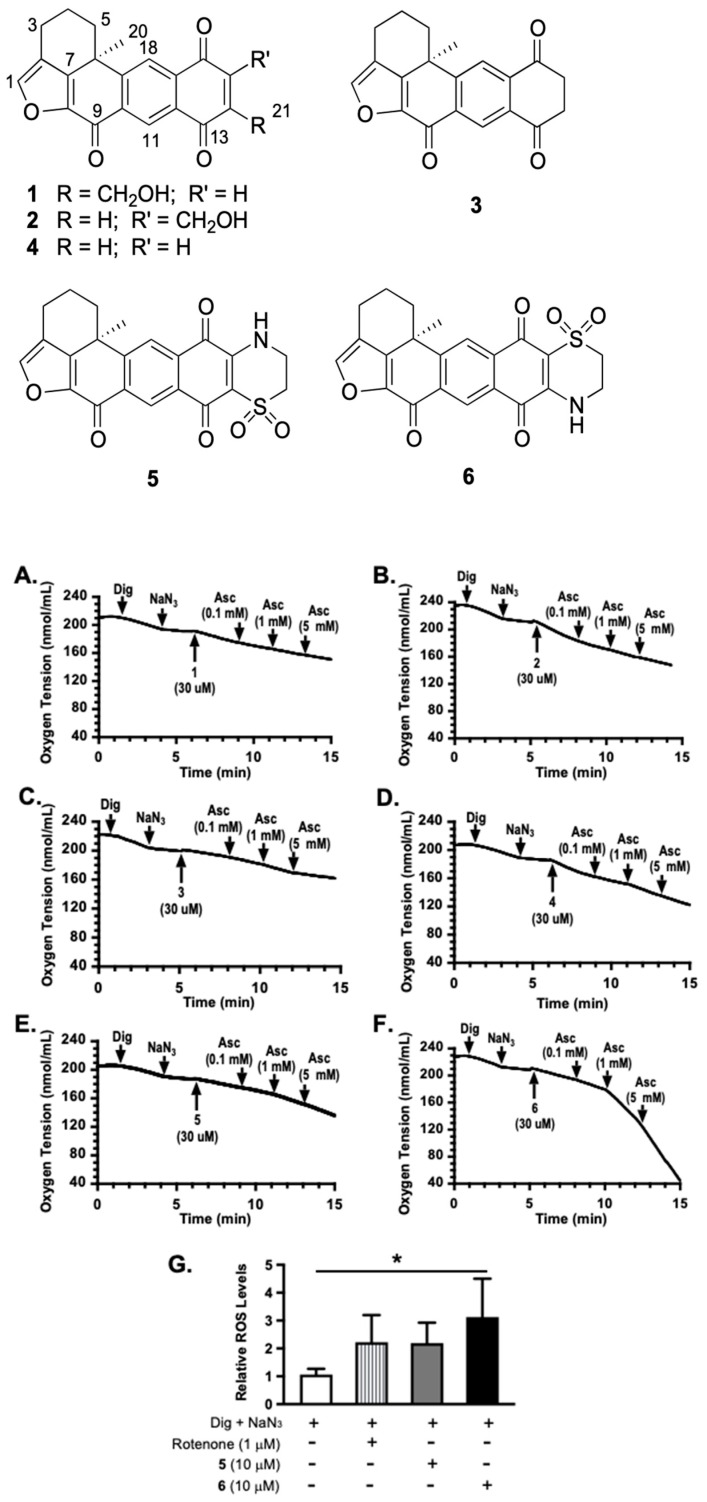
Xestoquinone derivatives stimulated sodium azide-stalled oxygen consumption and ascorbate enhanced adociaquinones’ activities. Oxygen consumption in digitonin (Dig)-permeabilized T47D cells was monitored in the absence of additional substrates. Compounds were added in a sequential manner at the specified time point (pointed arrow). (**A**) 14-hydroxymethylxestoquinone (**1**). (**B**) 15-hydroxymethylxestoquinone (**2**). (**C**) 14,15-dihydroxestoquinone (**3**). (**D**) Xestoquinone (**4**). (**E**) Adociaquinone A (**5**). (**F**) Adociaquinone B (**6**). Ascorbate (Asc) was added at incremental concentrations. Similar results were observed in independent experiments. (**G**) Levels of ROS in Dig-permeabilized T47D cells treated with specified compounds in the presence of NaN_3_. Data shown are mean + SD from two independent experiments (*n* = 6), using a dihydroethidium (DHE)-based method for measuring ROS. An asterisk “*” indicates statistically significant difference (*p* < 0.05).

To determine the mechanism of action for adociaquinones, oxygen consumption studies were performed in T47D cells. Digitonin (Dig) was applied to provide mitochondrial accessibility by permeabilizing the plasma membrane, and sodium azide (NaN_3_) was used to suppress mitochondrial oxygen consumption by inhibiting cytochrome c oxidase. As shown in Figure 1, 14-hydroxymethylxestoquinone (**1**, Figure 1A), 15-hydroxymethylxestoquinone (**2**, Figure 1B), 14,15-dihydroxestoquinone (**3**, Figure 1C), xestoquinone (**4**, Figure 1D), and adociaquinones A (**5**, Figure 1E) and B (**6**, Figure 1F) all restored NaN_3_-stalled oxygen consumption to various extents. Ascorbate (Asc) further enhanced adociaquinones-reinstated oxygen consumption in a concentration-dependent manner (Figure 1E,F). The combination of **6** and ascorbate yielded the most robust response. In a dihydroethidium (DHE)-based assay [8], **6** stimulated reactive oxygen species (ROS) production in Dig-permeabilized T47D cells in the presence of NaN_3_ (Figure 1G). These observations suggested that adociaquinones may function through redox cycling.

### 2.2. Tumor Cell Proliferation/Viability and Survival Studies

**Figure 2:**
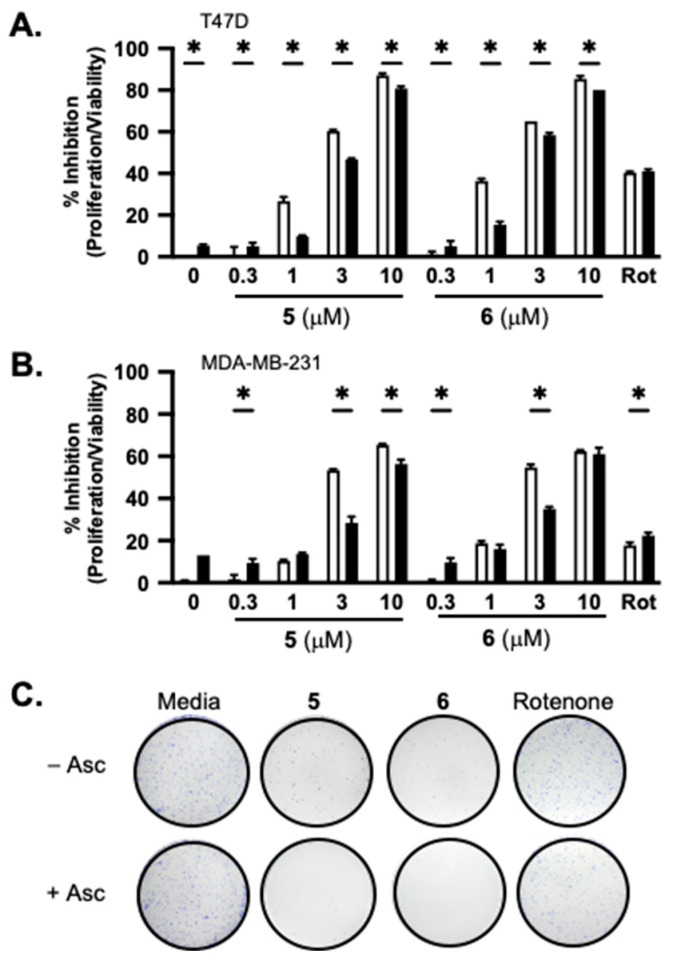
Ascorbate affects the activity of adociaquinones in a concentration- and time-dependent manner. Concentration-dependent effects of **5** and **6** on T47D (**A**) and MDA-MB-231 (**B**) cell proliferation/viability in the presence (solid bar) and absence (open bar) of ascorbate (1 mM). Rotenone (Rot, 1 μM) was included as a positive control. Cell proliferation/viability was determined in a standard SRB-based 48 h exposure assay and presented as “% Inhibition” of the untreated control. Data shown are average + SD (*n* = 3). An asterisk “*” indicates *p* < 0.05 for the with/without ascorbate pair comparison. (**C**) T47D cells were treated with combinations of specified compounds for 4 h, the conditioned media were removed, and the colonies formed after 10 days were fixed, stained, and photographed. The compounds used are ascorbate (Asc, 1 mM), **5** (10 μM), **6** (10 μM), and rotenone (1 μM).

Concentration–response studies were performed to further assess the effect of ascorbate on adociaquinones. Human breast cancer T47D and MDA-MB-231 cells served as in vitro models for estrogen-dependent early-stage and triple-negative late-stage diseases, respectively. In a standard 48 h SRB-based cell viability assay, **5** and **6** each suppressed cell proliferation/viability in a concentration-dependent manner. At lower concentrations, higher activities were observed in T47D cells (Figure 2A) compared to those in MDA-MB-231 cells (Figure 2B). While ascorbate (1 mM) exhibited modest inhibitory activity in both cell lines (5% in T47D cells and 13% in MDA-MB-231 cells), it reduced low-concentration adociaquinone-imposed growth inhibition [e.g., 1 μM of **5** and **6** in T47D (Figure 2A), and 3 μM of **5** and **6** in MDA-MB-231 (Figure 2B)]. One possible scenario is that adociaquinones exert cytostatic/cytotoxic effects through ROS, generated from redox cycling. Ascorbate reacts with ROS, ‘neutralizing’ those produced by low concentrations of adociaquinones but not by higher concentrations. The clonogenic survival assay measures the ability of tumor cells to form colonies. Four-hour exposure to **5** and **6** (10 μM each, respectively) drastically reduced the ability of T47D cells to form colonies, and ascorbate (1 mM) enhanced this suppression (Figure 2C).

### 2.3. Effects of Reducing Agents on Active Compounds Exerted HIF-1 Inhibition

**Figure 3:**
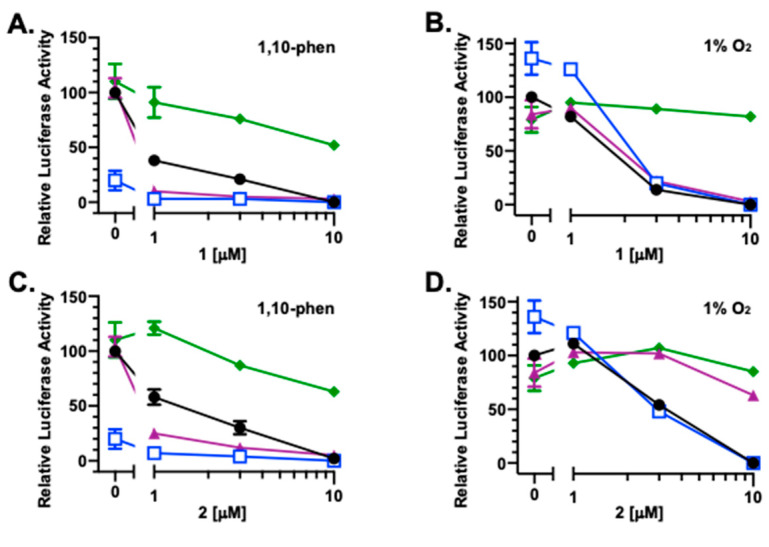

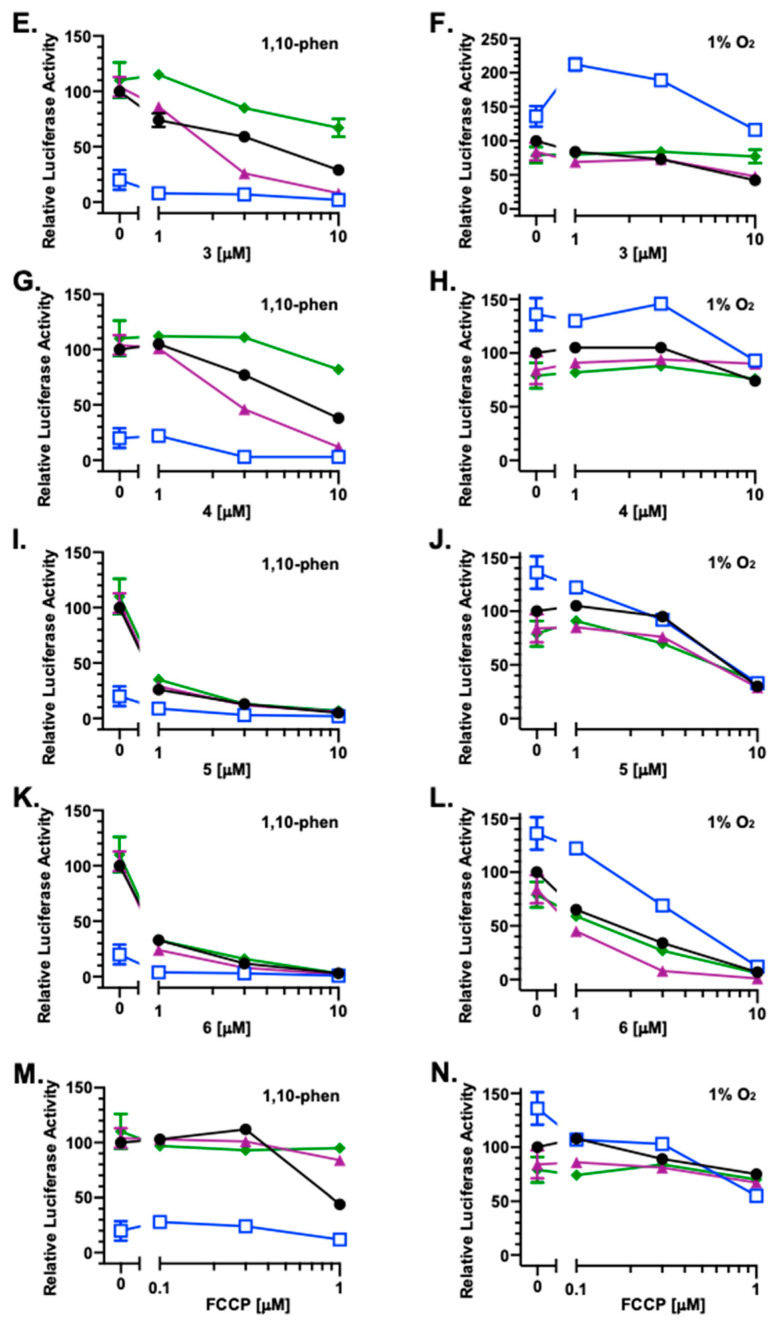
Inducing condition-dependent interference by ascorbate, NAC, and DTT on **1–6** and FCCP incurred HIF-1 inhibition. A T47D cell-based reporter assay was used to monitor HIF-1 activity. The inducing conditions are chemical hypoxia [10 μM of 1,10-phenanthroline; (**A**,**C**,**E**,**G**,**I**,**K**,**M**)] and hypoxia [1% O_2_; (**B**,**D**,**F**,**H**,**J**,**L**,**N**)], respectively. Compounds **1–6** and FCCP were tested at the specified concentrations in the absence (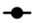) or presence of ascorbate (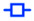), NAC (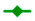), or DTT (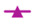). Luciferase activities are presented as a percentage of the induced control (1,10-phenanthroline or hypoxic media control, as appropriate). Data shown are averages ± standard deviation from two independent experiments (*n* = 12 for induced controls, ascorbate, NAC, and DTT; *n* = 3 for **1–6** and FCCP alone or in combination with the reducing agents).

To investigate the impact of redox homeostasis on the bioactivities of **1**–**6**, HIF-1 activity was measured in the presence and absence of commonly used antioxidants that include ascorbate, dithiothreitol (DTT), and *N*-acetylcysteine (NAC). In a T47D cell-based reporter assay, **1** (Figure 3A), **2** (Figure 3C), **3** (Figure 3E), **4** (Figure 3G), **5** (Figure 3I), **6** (Figure 3K), and the mitochondrial uncoupler FCCP (Figure 3M) each inhibited chemical hypoxia [10 μM of 1,10-phenanthroline (1,10-phen)]-induced HIF-1 activation, similar to that previously reported [4]. Ascorbate (1 mM) inhibited 1,10-phen-activated HIF-1 by 80% (Figure 3A). Combining ascorbate with **1**–**6** and FCCP yielded additive effects, inhibiting HIF-1 activation by chemical hypoxia (Figure 3A,C,E,G,I,K,M, respectively). Neither DTT (1 mM) nor NAC (1 mM) interfered with 1,10-phen-induced HIF-1 activation. The HIF-1 inhibitory activities displayed by **5** (Figure 3I) and **6** (Figure 3K) were not affected by DTT and NAC. However, DTT and NAC did decrease the inhibitory activity of FCCP on HIF-1 (Figure 3M). Further, DTT enhanced while NAC reduced the inhibition of 1,10-phen-activated HIF-1 produced by compounds **1**–**4** (Figure 3A,C,E,G).

Under hypoxic conditions (1% O_2_, 16 h), ascorbate enhanced HIF-1 activation by 36%, while neither DTT nor NAC exhibited pronounced effects (Figure 3B). For **1** and **2**, ascorbate did not affect their ability to inhibit hypoxia-induced HIF-1 activation, NAC suppressed the effects of both compounds, and DTT reduced **2**-imposed HIF-1 inhibition (Figure 3B,D). A concentration-dependent biphasic response was observed with the combination of ascorbate and **3** (Figure 3F). At lower concentrations, **3** amplified ascorbate-enhanced hypoxic HIF-1 activation. This enhancement was diminished at higher concentrations. Similar to that observed with **1**, NAC reduced **3**-exerted HIF inhibition and DTT did not. Under hypoxic conditions, **4** alone or in various combinations displayed only weak activities (Figure 3H). None of the three reducing agents interfered with the HIF-1 inhibition induced by **5** and **6** at the highest concentration (10 μM) (Figure 3J,L). However, ascorbate reduced while DTT enhanced the inhibitory activity of **6** at lower concentrations. At the concentrations tested, only modest effects were observed with FCCP, alone or in combination with the reducing agents (Figure 3N).

**Figure 4:**
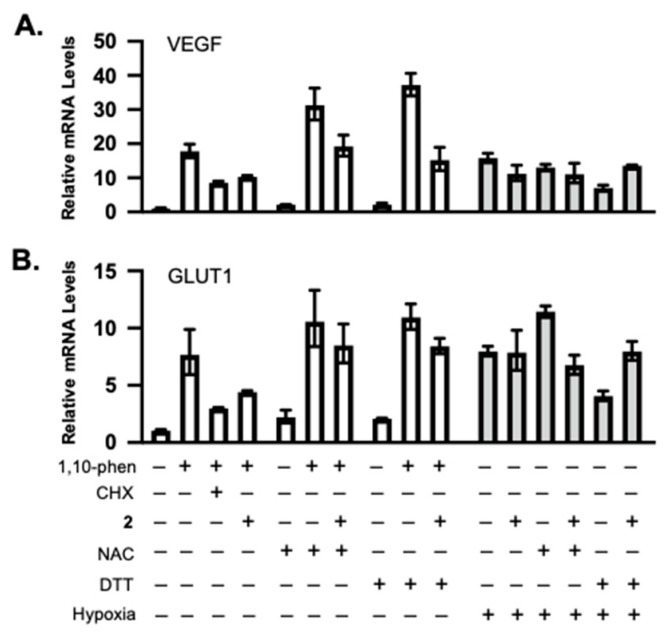
Compound **2** inhibited HIF-1 target gene expression, and this inhibitory activity was reduced by NAC and DTT. Real-time RT-PCR analysis of *VEGF* (**A**) and *GLUT1* (**B**) expression levels in total RNA samples prepared from T47D cells under specified conditions. Cycloheximide (CHX) inhibits protein synthesis and was included as a positive control. The relative mRNA levels (mean ± SD, *n* = 3) were determined by the ΔΔC_T_ method, normalized to an internal control (18S rRNA).

Because **2** was most affected by NAC and DTT (Figure 3C,D), it was selected as an example to further investigate the effect of thiol-reducing agents on the bioactivities of these xestoquinone derivatives. Exponentially grown T47D cells were exposed to **2** in the presence and absence of NAC or DTT. The incubation continued under HIF-1-inducing conditions—chemical hypoxia (10 μM of 1,10-phenanthroline) or hypoxia (1% O_2_), as appropriate. Following incubation, total RNA samples were prepared, and mRNA levels of HIF-1 target genes *VEGF* and *GLUT1* were determined by real-time RT-PCR (Figure 4A,B). The overall pattern of inhibition was similar to that observed in the reporter assay (Figure 3C,D). Specifically, 1,10-phenanthroline increased *VEGF* and *GLUT-1* mRNA expression, **2** inhibited this induction, and NAC overcame **2**-imposed inhibition (Figure 4). However, the extent of inhibition was reduced in the gene expression study in comparison to that observed in the reporter assay. The effects on hypoxia-induced *VEGF* and *GLUT-1* mRNAs were less pronounced.

### 2.4. Mechanistic Studies on the Signaling Pathways Affected by Active Compounds

**Figure 5:**
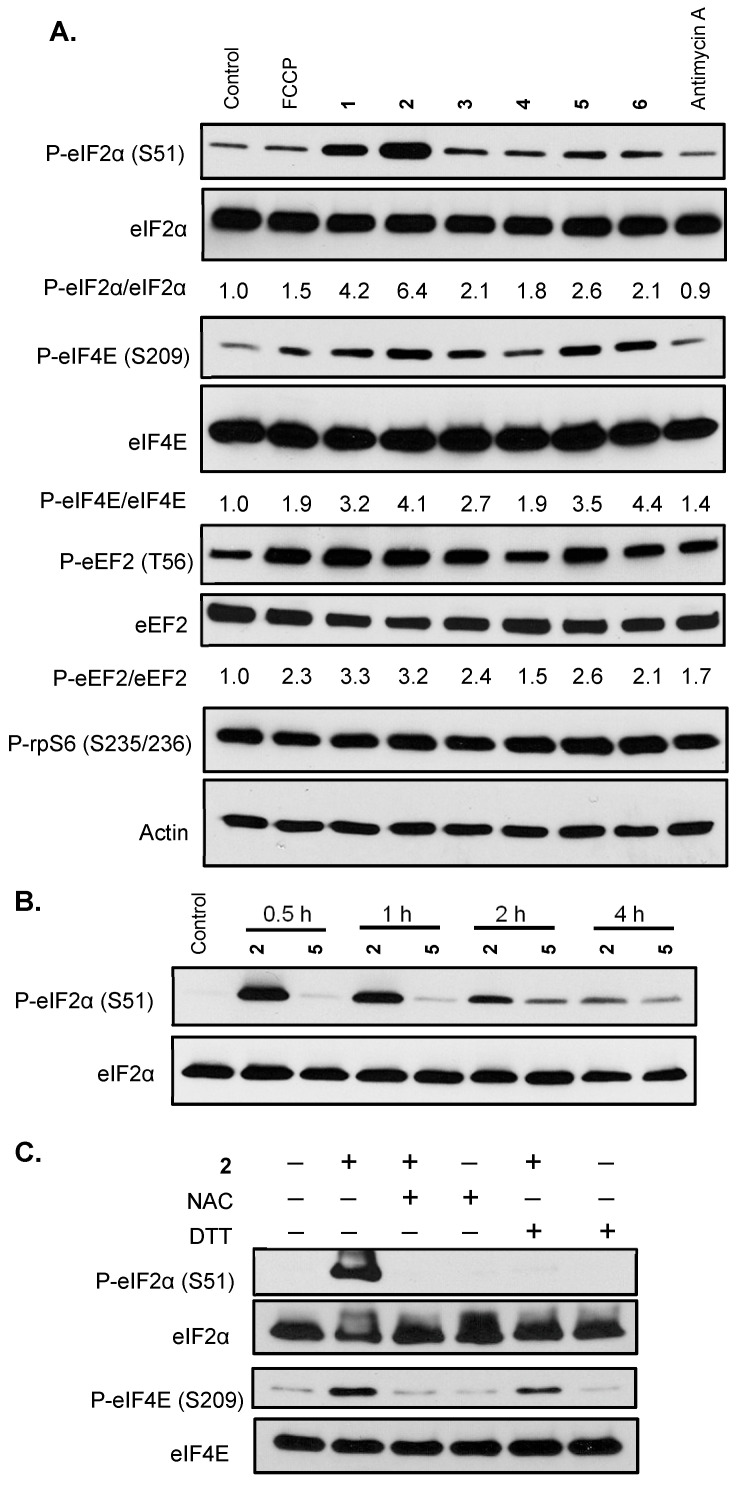
Xestoquinone derivatives and adociaquinones trigger cellular stress responses that inhibit protein translation. (**A**) Exponentially grown T47D cells were exposed to FCCP (0.3 μM), **1–6** each at 10 μM, and antimycin A (1 μM) for 30 min. Levels of phosphorylated eIF2α (P-eIF2α), total eIF2α (eIF2α), phosphorylated eIF4E (P-eIF4E), total eIF4E (eIF4E), phosphorylated eEF2 (P-eEF2), total eEF2 (eEF2), phosphorylated rpS6 (P-rpS6), and actin in the cell lysate samples were determined by Western blot. The ratio of phosphorylated protein/total protein was based on band intensity, quantified by Image J. Actin was included as a loading control. (**B**) T47D cells were treated with **2** (10 μM) and **5** (10 μM) for the specified time periods. Levels of phosphorylated and total eIF2α proteins were determined by Western blot. (**C**) T47D cells were exposed to **2** (10 μM) for 30 min in the presence or absence of NAC (1 mM) or DTT (1 mM). Levels of P-eIF2α, eIF2α, P-eIF4E, and eIF4E proteins were determined by Western blot.

A major consumer of energy, protein synthesis is tightly regulated and plays a pivotal role in cellular physiology. Compounds that interfere with oxygen and/or redox homeostasis often affect protein synthesis. The effects of **1–6** on representative signaling pathways that regulate translation were examined in T47D cells. Exponentially grown cells were treated with specified compounds for 30 min, cell lysate samples were prepared, and the levels of target proteins were determined by Western blot. One of the central mechanisms whereby cells respond to environmental stress is to phosphorylate the eukaryotic initiation factor 2 α subunit (eIF2α), preventing the formation of the eIF2-GTP-tRNAi^Met^ ternary complex, thus stalling the initiation of translation [9]. Compound **2** induced robust eIF2α phosphorylation, followed by **1**, a stereoisomer of **2** (Figure 5A). Compounds **3–6** also triggered eIF2α phosphorylation to a lesser degree (Figure 5A). A component of the eukaryotic translation initiation factor 4F (eIF4F) complex, eukaryotic initiation factor 4E (eIF4E) recognizes the 5′-7-methylguanine (m^7^G) cap. Phosphorylation of eIF4E contributes to the abandonment of translation initiation [10]. Compounds **6** and **2** incurred the most eIF4E phosphorylation, followed by **5** and **1**, **3**, and **4** (Figure 5A). Eukaryotic elongation factor 2 (eEF2) regulates translation elongation and eEF2 phosphorylation inhibits eEF2-dependent ribosomal translocation [11]. Compounds **1–6** induced eEF2 phosphorylation, with **1** and **2** exerting the most effects and **4** the least (Figure 5A). Evolutionarily conserved among eukaryotes, ribosomal protein S6 (rpS6) is a part of the 40S small ribosomal subunit that controls translation. The phosphorylation of rpS6 is considered an indicator for the activated PI3K/AKT/mTORC1 pathway [12]. As shown in Figure 5A, the levels of phosphorylated rpS6 proteins were not significantly affected by the test compounds. These results suggest that the active compounds elicited a cellular stress response that led to the inhibition of protein translation by phosphorylating key regulators, including eIF2α, eIF4E, and eEF2. Based on the eIF2α phosphorylation results, **2** and **5** were selected for a time-course study. Compound **2** exerted the greatest effect on eIF2α at 30 min and the signal intensity decreased over time (Figure 5B). Meanwhile, **5** induced the most eIF2α phosphorylation at 2 h (Figure 5B). Further mechanistic investigation revealed that **2**-induced eIF2α was blocked by NAC and DTT, respectively (Figure 5C). Similar inhibitory effects on **2**-induced eIF4E phosphorylation were observed in the presence of NAC and DTT (Figure 5C). In general, NAC was more effective than DTT at suppressing the function of **2**.

## 3. Discussion

**Figure 6:**
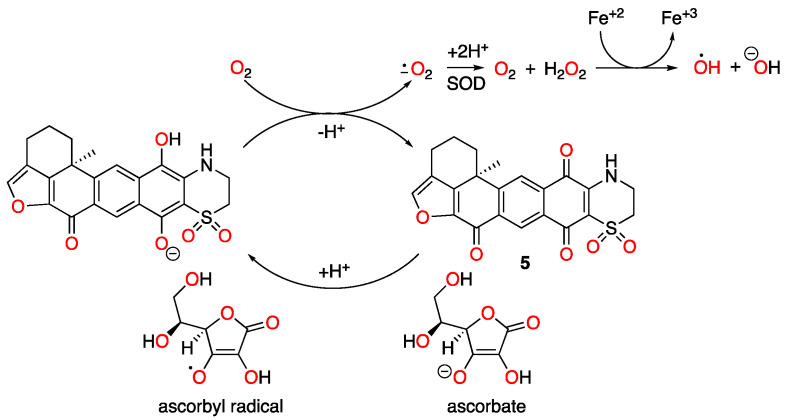
Proposed redox cycling scheme for adociaquinone A (**5**).

Natural products have been a major source of new drugs for centuries. Technological advancements have greatly expanded the capacity to discover bioactive natural products from marine organisms, including many that target specific signaling pathway(s)/molecule(s) critical for malignant progression [13,14,15]. Using a human breast cancer cell line-based platform, we have discovered chemically and mechanistically diverse agents that inhibit HIF-1, an important molecular target for anticancer drug discovery. Our previous research revealed that **1** decreased the mitochondrial membrane potential and **5** did not, although both enhanced cellular respiration [4]. Compound **2** exhibited an activity profile similar to that displayed by the structurally related **1**. The same type of correlation was observed between **5** and **6**. To discern the mechanism of action for adociaquinones, we tested the hypothesis that they may function through redox cycling. Mitochondria consume over 90% of cellular oxygen, primarily through oxidative phosphorylation. Sodium azide suppresses mitochondrial oxygen consumption by inhibiting ETC complex IV (cytochrome c oxidase). The fact that **5** and **6** each overcame sodium azide-imposed inhibition on respiration suggests that they stimulated non-mitochondrial oxygen consumption. Moreover, ascorbate enhanced this restoration in a concentration-dependent manner, implicating the involvement of ascorbate-facilitated redox cycling for adociaquinones (Figure 6). The observation that **6** (Figure 1F) was more active than **5** (Figure 1E) at stimulating oxygen consumption and ROS generation (Figure 1G) suggests that this reaction may be catalyzed by an enzyme(s) that is stereospecific. In a standard 48 h exposure study, ascorbate reduced the inhibition on cell proliferation/viability exerted by lower concentrations of adociaquinones (e.g., 1 μM in T47D cells and 3 μM in MDA-MB-231 cells, Figure 2A,B). One possible explanation is that the excessive amount of ascorbate reduces the ROS generated by a low concentration of adociaquinones, thus decreasing ROS-induced cytotoxicity. This ‘negative’ effect of ascorbate disappeared when adociaquinone concentrations increased (Figure 2A,B); presumably, the amount of ROS induced by adociaquinones overpowered the ROS scavenging capacity of ascorbate. However, ascorbate did enhance the inhibitory activities of adociaquinones A (**5**) and B (**6**) in the T47D cell-based clonogenic survival assay. It is possible that a shorter exposure time (4 h vs. 48 h) generates more ROS through ascorbate-facilitated redox cycling and cells at a lower density are more prone to ROS-incurred damage.

The canonical pathway for oxygen-dependent HIF-1 regulation includes prolyl hydroxylation, which tags the HIF-1α subunit for subsequent ubiquitination and proteasomal degradation [16,17]. Prolyl hydroxylases are dioxygenases that require iron and 2-oxoglutarate. Ascorbate facilitated HIF-1α degradation by promoting prolyl hydroxylase activity, especially in an oxygenated environment [18,19]. Thus, it is not surprising that ascorbate inhibited 1,10-phenanthroline-induced HIF activation when combined with **1–6** and FCCP (Figure 3A,C,E,G,I,K,M), respectively. However, ascorbate enhanced hypoxia-induced HIF activation, and the addition of **3** further induced this stimulation (Figure 3F). Because ascorbate can act as a pro-oxidant at high concentrations [20], and **3** is the most oxidized among the compounds tested, one possible explanation is that ascorbate and **3** inhibit HIF prolyl hydroxylase by oxidizing iron-(II) to iron-(III), further enhancing hypoxia-suppressed HIF-1α degradation and subsequent HIF-1 activation.

Mechanistic investigation revealed that **1–6** triggered cellular stress responses that block protein translation by phosphorylating key regulatory factors that include eIF2α, eIF4E, and eEF2. A time-course study that monitored the phosphorylation of eIF2α in the presence of **2** and **5** identified the difference in response time—**2** peaked at 30 min and the signal decayed over time, while **5** peaked at 2 h (Figure 5B). These results suggest that the structurally related **1** and **2** may function through similar mechanisms, which are different from the structurally related **5** and **6**. A recent study discovered that the monosubstituted xestoquinone derivatives **1** and **2**, but not the disubstituted **5** and **6**, activated the oxidative stress-responsive nuclear factor erythroid 2-related factor 2 (Nrf2) in an MCF-7 cell-based reporter assay [21]. The observation that NAC and DTT each blocked the **2**-induced phosphorylation of eIF2α and eIF4E (Figure 5C) suggested that the enzyme(s) targeted by **2** may contain a cysteine residue at the active site. This may contribute to the enhanced ‘reversal’ activity observed with NAC in comparison to DTT. This speculation is supported by earlier reports that sulfhydryl modification mediates xestoquinone (**4**)-associated inotropic action [22,23].

While cell lines have served as important tools in cancer research and drug discovery, factors such as genetic heterogeneity, cell–cell interaction, the microenvironment, the metabolic state, etc., can influence how cancer cells respond to drugs/chemicals. Thus, this established cancer cell line-based study is limited in scope. However, the findings that the activities of these xestoquinone derivatives are altered by ascorbate, DTT, and NAC are significant. A number of chemotherapeutic drugs exert their anticancer effects through the generation of ROS. It warrants further investigation to assess the effects of commonly consumed antioxidants and supplements on the efficacy of cancer drugs, especially those that function through stimulating ROS generation.

## 4. Materials and Methods

### 4.1. Cell Lines and Chemicals

Human breast cancer T47D and MDA-MB-231 cells were purchased from ATCC (Manassas, VA, USA). Cells were maintained in DMEM/F12 media (Corning, NY, USA), supplemented with FBS (10%, *v*/*v*, ThermoFisher Scientific, Waltham, MA, USA) and a mixture of Penn/Strep (1×, ThermoFisher Scientific, Waltham, MA, USA) at 37 °C under 5% CO_2_/95% air, in a humidified environment. Compounds were isolated and characterized previously [4]. The purities of all compounds were judged based on the percentage of the integrated signal at UV 220 nm. Compounds **1**–**6**, submitted for bioassay, were at least 95% pure as judged by this method [4]. All other chemicals and solvents were purchased from Millipore-Sigma (Darmstadt, Germany).

### 4.2. T47D Cell-Based Respiration Studies and ROS Measurement

The T47D cell-based respiration assay was performed as described [24], with the modification that NaN_3_ was added at 10 mM to inhibit mitochondrial oxygen consumption. For ROS measurement, T47D cells were plated at the density of 30,000 cells/well into black 96-well cell culture plates with a clear bottom (Greiner Bio-One, Kremsmünster, Austria). Following incubation overnight at 37 °C, the cells were washed twice with 1× DPBS (Millipore-Sigma, Darmstadt, Germany). Freshly prepared dihydroethidium (DHE, ThermoFisher Scientific, MA, USA) was added at the concentration of 25 μM, and the incubation continued for 30 min. The cells were washed twice with 1× DPBS (Millipore-Sigma, Darmstadt, Germany), compounds were added at the specified concentrations, incubated at 37 °C for 30 min, and fluorescence was measured with 485 nm (ex)/620 nm (em) on a Bio-Tek Synergy plate reader.

### 4.3. Cell Proliferation/Viability and Clonogenic Survival Assays

Exponentially grown T47D and MDA-MB-231 cells were plated at the density of 30,000 cells/well into 96-well cell culture plates (Greiner Bio-One, Kremsmünster, Austria) in a volume of 100 μL of complete culture media, and incubated overnight at 37 °C. Compounds were diluted from stock solutions and added to the cells in a volume of 100 μL of serum-free media/well to achieve the specified concentrations. The incubation continued for 48 h and cell viability was determined by the SRB-based method [25]. The following formula was used for data processing: % Inhibition = 100 × (1 − OD_490 treated_/OD_490 media_).

For the clonogenic survival assay, T47D cells were seeded at the density of 3000 cells/well into 6-well cell culture plates (Greiner Bio-One, Kremsmünster, Austria). After overnight incubation at 37 °C, the cells were exposed to test compounds at the specified concentrations in DMEM/F12, supplemented with 5% (*v*/*v*) FBS and 1× Penn/Strep, for 4 h. The conditioned media were replaced with complete media and the incubation continued at 37 °C for 9 days, with fresh media changed after 5 days. The cells were fixed with cold methanol at 4 °C for 5 min, stained with 0.1% (*w*/*v*) crystal violet (Millipore-Sigma, Darmstadt, Germany) in 1× PBS at room temperature for 30 min, washed twice with water, air dried, and photographed.

### 4.4. Cell-Based Reporter Assay and Quantitative Real-Time RT-PCR

A T47D cell-based reporter assay that measures HIF-1 activity was performed as described [26]. To quantify the levels of *VEGF* and *GLUT1* mRNA, T47D cells (0.9 × 10^6^ per well) were plated into 6-well cell culture plates (Greiner Bio-One, Kremsmünster, Austria), incubated overnight, and compounds were added in serum-free medium (final FBS, 5%, *v*/*v*). The final concentrations were 10 μM **2**, 1 mM NAC, 1 mM DTT, and 1 μM cycloheximide (CHX). After an initial 30 min period, the exposure continued for another 16 h in the presence of 10 μM of 1,10-phenanthroline (1,10-phen) under 5% CO_2_/95% air, or under 5% CO_2_/1% O_2_/ 94% N_2_ (hypoxia) at 37 °C. Following treatments, total RNA samples were prepared with the RNeasy Mini Kit (QIAGEN, Hilden, Germany), first strand cDNAs were synthesized, quantitative real-time PCR reactions were performed with *VEGF-*, *GLUT1-*, and *18S rRNA*-specific primers, and data were analyzed as described [26].

### 4.5. Signaling-Related Western Blot Analysis

Plating of exponentially grown T47D cells, compound treatments, cell lysate preparation, protein concentration determination, SDS-PAGE, and Western blot analysis were performed as described [27]. The following compounds were added at the specified concentrations: FCCP (0.3 μM), **1**–**6** each at 10 μM, antimycin A (1 μM), NAC (1 mM), and DTT (1 mM). Unless specified, a 30 min exposure time was applied for signaling studies. For Western blot, the following primary antibodies were purchased from Cell Signaling Technology (Danvers, MA, USA) and used according to the manufacturer’s instructions: phospho-eIF2α (Ser51) (#9721), eIF2α (#9722), phospho-eIF4E (Ser209) (#9741), eIF4E (#9742), phospho-eEF2 (Thr56) (#2331), eEF2 (#2332), and phospho-S6 ribosomal protein (Ser235/236) (#2211). The anti-actin antibody was from PhosphoSolutions (Aurora, CO, USA, #125-ACT) and used at a 1:5000 dilution.

### 4.6. Statistical Analyses

GraphPad Prism 8 (Dotmatics, MA, USA) was used for data processing, presentation, and comparison. Differences were considered statistically significant when *p* < 0.05, acquired from one-way ANOVA followed by Bonferroni post hoc analyses.

## 5. Conclusions

While xestoquinone derivatives such as 14-hydroxymethylxestoquinone stimulate cellular respiration by dissipating the mitochondria membrane potential, adociaquinones enhance oxygen consumption through redox cycling. Ascorbate decreased the growth inhibitory activity exhibited by adociaquinones at lower concentrations, and increased the cytotoxicity of higher concentrations of adociaquinones. Reducing agents (ascorbate, NAC, and DTT) interfered with the HIF-1 inhibitory activities displayed by **1**–**6** in an inducing condition-dependent manner. Mechanistic investigation revealed that **1**–**6** triggered cellular stress responses that stalled protein translation by phosphorylating initiation factors eIF2α and eIF4E, and elongation factor eEF2. The thiol-reducing agent *N*-acetylcysteine blocked the most active 15-hydroxymethylxestoquinone (**2**), implicating sulfhydryl modification as a potential mechanism of action.

## Data Availability

Data are contained within the article. Further inquiries can be directed to the corresponding author.
